# MS Spasticity: Take Control (STC) for ambulatory adults: protocol for a randomized controlled trial

**DOI:** 10.1186/s12883-020-01902-1

**Published:** 2020-10-07

**Authors:** Cinda L. Hugos, Michelle H. Cameron

**Affiliations:** 1 VA Portland Health Care System, 3710 SW US Veterans Hospital Rd. R&D 27, Portland, OR 97239 USA; 2 Department of Neurology, Oregon Health & Science University, 3303 SW Sam Jackson Park Rd. L226, Portland, OR 97239 USA

**Keywords:** Multiple sclerosis, Spasticity, Exercise, Stretching, Symptom management, Clinical trial, Randomized controlled trial, Self-management, Fatigue, Walking

## Abstract

**Background:**

Spasticity affects 60–80% of people with multiple sclerosis (MS), impacting activity, participation and quality of life. We developed the group delivered spasticity self-management program, “MS Spasticity: Take Control” (STC), with DVDs for education and lower extremity stretching. STC is based on an international guideline and recommendations from systematic reviews and emphasizes the importance of stretching with specific stretching exercises. Our pilot trial (*n* = 38) compared STC followed by one month of home stretching practice to unguided use of the National MS Society (NMSS) brochure titled “Stretching for People with MS: An Illustrated Manual,” also followed by one month of home stretching practice. In this pilot trial, STC showed promising effects on the impact of spasticity (MS Spasticity Scale-88) and other self-report and physical performance measures. We will now carry out a fully-powered trial to evaluate the effect of STC compared to a comparably delivered control program on the impact and severity of spasticity in people with MS and self-reported lower extremity spasticity.

**Methods:**

Two hundred-twenty ambulatory adults with MS self-reported spasticity interfering with daily activities will be randomized 1:1 to STC or control, using the same NMSS brochure used in the pilot study, with both programs delivered in groups with trained facilitators. Outcomes are the impact of spasticity with the MS Spasticity Scale-88, the severity of spasticity with the Numeric Rating Scale for Spasticity, other self-report questionnaires, and physical performance walking measures at baseline and one and 6 months after the interventions.

**Discussion:**

Stretching is the cornerstone of spasticity management. Stretching takes time and energy every day. Unfortunately, beyond the logical expectation that regular stretching should help prevent muscle shortening and contractures in the presence of spasticity, there is very little data on the effects of stretching on spasticity in people with MS or any other condition. Our pilot trial of STC suggested that education and stretching help reduce the impact of spasticity. To definitively determine if this education and instructional program with daily stretching practice is effective, a fully powered trial with a comparable control intervention and facilitators who did not create STC is needed. Here we report the protocol for this trial.

**Trial registration:**

NCT03166930 May 25, 2017.

## Background

Multiple sclerosis (MS) is a common and often disabling disease of the central nervous system that affects approximately 2.5 million people worldwide and over 900,000 people in the United States [1]. Spasticity occurs in 60–80% of people with MS (PwMS) contributing to MS disability with gait disorders, falls, fatigue, spasms, pain, and potentially hastening the time to wheelchair dependence. Increased disability and dependence can then lead to social isolation and depression, cardiovascular disease, muscle fibrosis and joint contracture with secondary skin breakdown, infection and death [2–4]. Spasticity is classically defined by Lance as “a motor disorder characterized by a velocity dependent increase in tonic stretch reflexes (muscle tone) with exaggerated tendon jerks, resulting from hyperexcitability of the stretch reflex, as one component of the upper motor neuron syndrome” [5]. A more physiologically based definition, from Pandyan et al., is “disordered sensori-motor control, resulting from an upper motor neurone lesion, presenting as intermittent or sustained involuntary activation of muscles” [6]. A more patient-centered description of spasticity from the North American Research Committee on MS (NARCOMS) is “unusual tightening of muscles that feels like leg stiffness, jumping of legs, a repetitive bouncing of the foot, muscle cramping in the legs or arms, the legs going out tight and straight or drawing up” [2].

Spasticity is managed with rehabilitation interventions, medications, procedures and complementary and alternative medical (CAM) approaches. Typically, spasticity management begins with education and exercise. Exercise, including range of motion, stretching, strengthening, and conditioning is often included in referral to skilled rehabilitation that may also include orthotics, positioning, prolonged static stretching with standing or serial casting and neuromuscular stimulation [3, 4]. The most common first line medications prescribed for spasticity management are baclofen and tizanidine, with gabapentin and benzodiazepines as second line medications, [2, 7, 8] with limited support for any of these and no one shown to be superior to any others [7]. The medical procedures most commonly used for spasticity management are injections of botulinum toxin for local spasticity and surgical implantation of intrathecal baclofen pumps for severe, intractable, generalized spasticity [3, 4, 7, 8]. CAM approaches include massage, acupuncture and cannabinoids. There is high quality evidence that certain cannabinoid preparations reduce patient-reported spasticity in PwMS and these are recommended as add-on medications for treatment resistant spasticity where they are legal [9–11]. However, no tetrahydrocannabinol (THC) containing cannabis-derived medication has been approved by the Food and Drug Administration (FDA) in the United States and, therefore, in the United States patients only have access to unregulated cannabis preparations even in states where medical marijuana has been legalized.

Progress in the rehabilitation management of MS-related spasticity has been stalled for over 20 years for a number of reasons [12]. Measurement of spasticity has continued to rely on the Ashworth scale (AS) and the Modified Ashworth Scale (MAS), but these measures have substantial limitations. Both rely on subjective reporting of an examiner’s rating of only one component of spasticity, the response of tissue to quick stretch applied by the examiner, failing to capture the impact or severity of spasticity from the patient’s perspective [13–18]. There is no compelling evidence that stretching of muscles and other tissues, the recommended foundational intervention prescribed for all people with spasticity, is effective for people with spasticity caused by MS or other conditions [19–22]. Systematic reviews found limited and inconclusive evidence of benefit for stretching for spasticity management and no benefit for contracture prevention and management up to seven months and no information beyond seven months [20–22]. Only one small study with 30 PwMS and spasticity in the quadriceps found significant improvement on the MAS with oral baclofen compared to placebo or to placebo with stretching exercises. Adding stretching exercises to baclofen treatment was associated with a trend for additional benefit [19]. Meanwhile, stretching remains the recommended cornerstone of spasticity management, from the earliest symptoms of stiffness through the onset of severe disability with contractures and deformities [3, 4, 12, 20–22]. There has been no standardized program of stretching to study [7, 12, 20] so stretching instruction has relied on one-on-one physical therapy or unguided use of written materials.

In response to the Spasticity Management in MS clinical practice guideline, criticisms of the AS and MAS, and need for validated patient reported outcome measures for the National Institutes of Health, the Department of Defense, the FDA and others, patient-centered measures of the impact and severity of spasticity were developed. These include the MS Spasticity Scale-88 (MSSS), an 88-item questionnaire of the impact of spasticity, and the Numeric Rating Scale for Spasticity (NRSs), an eleven-point severity rating scale [23, 24].

Hugos and a colleague created the MS Spasticity: Take Control program (STC), a self-management program of education and standardized stretching exercises for daily practice to manage MS spasticity, with DVDs and manuals based on the guideline [25]. While standardized, STC provides variations of the stretches for individual customization to meet the needs and preferences of participants. We tested STC in a pilot randomized controlled trial with 38 PwMS and self-reported spasticity randomized 1:1 to STC followed by one month of home stretching practice or to the control condition of unguided use of a brochure from the National MS Society (NMSS), “Stretching for People with MS: An Illustrated Manual,” also followed by one month of home stretching practice [26]. STC was facilitated by Hugos, the study PI and the creator of the STC program. The trial demonstrated feasibility and acceptance of STC with enrollment of 40 subjects in three months and 95% retention with outcome data on 38 subjects and an initial signal of efficacy. Between baseline and follow-up, mean MSSS Total scores (STC − 27.8, control − 3.7, *p* < 0.03), mean MSSS Pain and Discomfort subscale scores (STC − 3.9, control + 0.3, *p* < 0.02) and mean MSSS Muscle Spasms subscale scores (STC − 5.0, control − 0.8, *p* < 0.03) improved significantly more in the STC group than in the control group [25]. The current unmet need is for testing of STC compared to a control intervention matched for time and attention in a fully powered trial with facilitators who did not create the program. This trial is described here. Our long-term goal is to determine if the STC program of education and daily stretching improves quality of life and functional outcomes in PwMS. Below we describe the aims and associated hypotheses of the study, outcome measures, and the data analysis plan.

## Methods/design

### Specific aims

#### Specific aim #1

The first aim of this study is to compare the effects of STC to a comparably delivered control program on the impact and severity of spasticity, other patient reported outcomes, and functional walking tests in ambulatory adults with MS and self-reported spasticity after one month of home daily stretching participation. The primary outcome of this study will be the impact of spasticity as measured by the MS Spasticity Scale-88 (MSSS) and a secondary outcome will be the severity of spasticity as measured by the Numeric Rating Scale for Spasticity (NRSs) after one month of home stretching practice [23, 24]. Other secondary outcomes will be fatigue measured with the Modified Fatigue Impact Scale (MFIS) [27], Emotional Distress/Depression measured with the PROMIS Short Form 8a [28], sleep quality measured with the Pittsburgh Sleep Quality Index (PSQI) [29], impact of MS on daily life measured with the MS Impact Scale-29 (MSIS) [30], impact of MS on walking measured with the MS Walking Scale-12 (MSWS) [31], timed walking measured with the Timed Up and Go (TUG) [32] and the Timed 25 Foot Walk (T25FW) [33], and frequency of stretching for MS lower extremity spasticity measured with daily diaries. Our hypothesis is that the STC education and exercise program, in conjunction with home practice, will reduce the impact of spasticity more than the comparable control program.

#### Specific aim #2

This aim is to determine if the effects of STC are sustained for six months. We hypothesize that differences in the effects of STC and the control program on the impact and severity of spasticity, other patient reported outcomes, timed walking tests, and participation in the stretching exercises will be sustained at six months. This will provide information about feasibility of continuing the daily exercises for six months and durability of effects on the above measures.

Here we describe the full-scale parallel group superiority trial comparing STC to a similar active control. It is expected that this study will inform us if the STC program with daily home stretching is beneficial in the short-term - after one month of home stretching, and in the medium-term - after six months of home stretching, compared to control.

### Design

This is a randomized controlled parallel treatment trial with two arms, with both arms consisting of a baseline visit, 2 classes approximately one week apart and 2 outcome visits 1 and 6 months after the classes (Table 1). Two hundred-twenty ambulatory adults (projecting for 10% drop out) with MS and self-reported lower extremity spasticity that interferes with their daily activities including sleep will be recruited (110 per arm) from the VA Portland Health Care System (VAPORHCS) and Oregon Health & Science University (OHSU) MS Clinics and the surrounding community in Portland, Oregon. Participants meeting the inclusion/exclusion criteria undergo a baseline assessment and then are randomized to receive either STC or the comparably delivered control program using the NMSS brochure “*Stretching for People with MS: An Illustrated Manual”* [26]. The study statistician generates the randomization lists. The study coordinator puts the randomized group assignments in sequentially numbered envelopes. When the baseline visit is completed, the participant is given the next unassigned envelope in the sequence. Men are randomized separately from women.

**Table 1:** Sequence of study activities and visit durations

Visit/Activity	Baseline Visit	Randomization to STC or control	Class 1	Class 2	Outcome Visit 1	Outcome Visit 2	Home exercises and record in daily diaries
Timing	Up to 2 months before Class 1	After Baseline visit	Week 1	Week 2	One month after Class 2	Six months after Class 2	After Class 2 through 6 months
Consent (20–30 min)	X						
Walking measures (5 min)	X				X	X	
Questionnaires (60–90 min)	X				X	X	
Active or control program of education and exercise instruction			X	X			
Total time	1 ½-2 h		2 h	2 h	1–1½ hours	1–1½ hours	15–20 min/day

### Participants

Adults with MS and self-reported spasticity are recruited from NW Oregon and SW Washington (within approximately a 50-mile radius of Portland) by personal contact in the VAPORHCS and OHSU MS Center clinics; posted flyers advertising the study; emails sent from the OHSU electronic medical record system (EMR); personalized letters to OHSU patients who have not opted into EMR messaging; postings on VA, OHSU and NMSS websites; newsletters; email blasts from the NMSS and advertisements in local media, if necessary. Both men and women will be recruited from the VA MS Clinic. Only women will be recruited from other sources because the study sponsor restricts male participation to Veterans only. Potential participants are screened during a clinic visit or by phone to verify they meet study eligibility criteria and a definite diagnosis of MS has been confirmed by a neurologist.

### Inclusion and exclusion criteria

#### Inclusion criteria

- A diagnosis of MS by 2017 McDonald Criteria [34]
- At least 18 years of age
- Able to walk 25 ft with or without any assistive devices (Patient Determined Disease Steps (PDDS) < 7) [35]
- Fluent in written and spoken English, as materials are not validated in languages other than English
- Presence of self-reported lower extremity spasticity interfering with daily activities using the NARCOMS definition of spasticity [2]
- Participants may have any subtype of MS and be on any medications or no medications.

#### Exclusion criteria

- Any uncontrolled medical or mental condition that, in the opinion of the PI, would limit participation or completion of the study
- Any self-reported neurological condition other than MS that is known to cause spasticity
- Participation in other interventional research studies.

Participants may be on any medications or no medications. We request participants not change their medications during the study unless absolutely necessary. MS disease modifying medications and spasticity management medications are collected at baseline and updated again at the one and six month outcome visits for analysis that any changes may have on the study results.

### Baseline visit

#### Consent

At the time of subject enrollment, an approved study team member reviews the informed consent form approved by the joint Veterans Administration Portland Health Care System and Oregon Health & Science University Institutional Review Board number IRB00000471, named Oregon Hlth & Science U IRB #3, with participants and then signs as witness.

#### Baseline questionnaires

Participants then complete paper questionnaires (approximately 60–90 min with the demographic, medical history, medication, and outcome questionnaires – see Outcomes visits for these questionnaires) and timed walking tests. The demographics collected are gender, age, education, ethnicity, employment status, clinical subtype of MS, marital status, year of MS diagnosis, past/concurrent medical history, medications, past and current cannabis use, and military service-connection. Patient Determined Disease Steps (PDDS) determine level of disability. These measures take approximately 30 min to complete. Participants are asked to not change their disease modifying medications or spasticity management medications through the duration of the study unless absolutely necessary.

#### Randomization

Participants are then randomized 1:1 to either STC or control. To optimize blinding, participants are not told which group they are assigned, but just told the day and time of their next visit which corresponds to the appropriately assigned program.

### Interventions

This study compares STC to a control intervention of similar duration, format and contact with a facilitator and other PwMS in groups of three to 12 people. Both interventions are delivered in two 2-h group classes approximately one week apart and facilitated by trained facilitators who are not subject-matter experts. All relevant concomitant care is allowed.

All participants are given a yoga mat so they can perform exercises lying on the floor since there are no beds or mat tables for the exercises in VA meeting rooms. None of the exercises must be done on the floor. Participants are told up front these exercises do not require getting on/off the floor which may be a deterrent to participation for people with spasticity and/or weakness and limited mobility. The mats only provide an option for trying the exercises on the floor in the group classes and as an option for their home exercise. All participants are given a strap to facilitate completion of the exercise options that use a strap. Participants receive reminder calls and/or emails one to two workdays before each scheduled visit. Attendance is taken at each class by the facilitator and/or a research assistant. The first participants were enrolled in September 2018. Enrollment is expected to continue through 2020.

#### Active intervention

MS Spasticity Take Control (STC) [25] was created as an implementation and self-management program for the Spasticity Management in MS clinical practice guideline based on the team’s experience doing the same with the Fatigue and MS clinical practice guideline [36–38].

STC consists of two 2-h classes one week apart. The first class of STC has introductions, an icebreaker activity, a review of group rules for an optimal experience for all, viewing and discussing the education DVD, a 10-min break, viewing and discussing the lower extremity stretching DVD, and preparing for the next class of group stretching instruction. In the second class, participants practice all the stretches. The goal of the second class is to find at least one exercise for each of eight lower body areas for a daily 15- to 20-min stretching routine. Facilitator and participant manuals are used to guide the program and provide reference material for home use and the stretching exercises. Participants are instructed to begin their daily home practice the next day with the help of the stretching DVD and/or photos of the stretches in the manuals, if needed, and start recording their practice in the daily diaries provided.

STC assets include the following:

a) A 28-min DVD with education and information about MS-related spasticity features MS professionals and poignant stories by PwMS and includes the following topics:

- recognizing spasticity;
- quality of life and the importance of behaviors and tools to maintain independence and participation;
- recognizing spasticity triggers;
- treatment of mild spasticity with stretching and other exercises;
- treatment of mild to moderate spasticity with medications, stretching, and other exercises;
- treatment of moderate to severe spasticity with botulinum toxin injections and/or intrathecal baclofen pump implantation in addition to stretching and possibly medications. This program does not address other possible surgeries for treatment of severe intractable spasticity. Ablative and/or orthopedic surgical procedures were beyond the scope of this program for mild to moderate spasticity for ambulatory PwMS and spasticity;
- treatment of mild/moderate/severe spasticity with complementary and alternative treatments (relaxation, massage, acupuncture, meditation, yoga, and different diets, but excluding cannabinoids),
- and a message about the importance of taking control of MS spasticity for overall health and quality of life.

b) A 20-min lower extremity stretching DVD teaches a standardized, yet individualized, program of daily stretching for MS-related spasticity. Eight body areas are targeted and several alternatives positions are provided for the stretches with the goal of each person finding at least one exercise in each group they can do for an approximately 15- to 20-min per day stretching routine. The exercises are:

- ‘elongate’ the whole body as a preparatory relaxation exercise;
- two choices each for trunk rotation, inner thighs, upper calves, lower calves, and hip flexors/extensors;
- seven choices for hamstring,
- and eight choices for quadriceps.

The choices provide positioning options for comfort and ease of completing the exercises and to allow easier or more challenging stretches. See Table 2 for muscle groups and positioning. The options are not designed to be a progression although some could be considered progressions with positions that are easier initially for tight muscles and then changing to other positions as the muscles become more flexible and the person becomes familiar with the exercises. Likewise, easier options are available if MS worsens. The exercises will naturally be progressive if the person reaches the same level of intensity but is able to move further. Any one of the exercises in each group is sufficient to provide an adequate stretch when applied as instructed, in nearly anyone with MS of any ambulatory ability. For instance, there are hamstring and quadriceps stretches lying down and sitting that do not challenge balance. Standing options for hamstrings and quadriceps may challenge balance excessively for some, increasing risk of falls so would not be appropriate. The standing calf stretches are the same positions that MS participants preferred in a recent study measuring torque applied during plantar flexor stretches that provided a strong but safe stretch. In the same study, the sitting calf stretch was found to be the stretch that PwMS could hold the longest (5 min sitting compared to 2.5 min standing), both exceeding the 30–60 s recommended in STC indicating participants should be able to meet the 30–60 s STC recommendations [39].

**Table 2:** MS Spasticity: Take Control Stretches

	Positions		
Body areas/Muscle groups	Lying	Sitting	Standing
Whole body elongation	X		
Trunk rotation	X	X	
Inner thighs	X	X	
Hip flexors/extensors	X		
Hamstrings	X	X	X
Quadriceps	X	X	X
Upper calves		X	X
Lower calves		X	X

c) Participant manuals include written material with all the information in the first DVD, learning objectives, a glossary of terms, space for notes and personal reflections, and still photos with written instructions for all the stretches included in the second DVD.

d) Facilitator manuals help with organizing and leading the classes. In addition to the information in the participant manuals, the facilitator manuals have agendas and tips to keep the group classes running smoothly.

STC includes the recommendations for a standardized program from the 2008 systematic review on stretching for spasticity [20]. Participants are instructed as follows in the DVD, manuals, and group classes: “Keep breathing and do not hold your breath when stretching. Drink plenty of water throughout the day. When you stretch, you should feel a gentle pull but no pain in the muscles being stretched. Proceed slowly until you feel the gentle pull. Pain is a protective response to overstretching. On a scale of 0-10, people new to stretching should stretch in the 2-3/10 range, specifically feeling a pull in the tight muscle but no pain. Once you are familiar with your own reaction to stretching, you may find you are able to stretch in the 4-6/10 range for more benefit. Muscles need to relax to get fully lengthened. A stretch should last for 30 to 60 seconds. Stretching needs to be done at least once every day. Some people find stretching several times a day keeps them more comfortable and able to move more freely. Set your personal stretching goal.” The classes display a poster as a reminder with the following: “Stretching should: Be done daily. Not be painful. Be held for a minimum of 30 to 60 seconds.” Specifically, per the recommendations for a standardized program [20]:

- intensity is the “2-3/10 range and eventually the 4-6/10 range” for “a gentle pull but no pain”;
- velocity is “proceed slowly until you feel a gentle pull”;
- repetitions are “at least once per day”, that is at least one repetition one time per day;
- duration is “30–60 s”,
- frequency is “daily”.

The following options for positioning are included: lying (elongate, trunk rotation, inner thighs, hip flexors/extensors, hamstrings and quadriceps), sitting (trunk rotation, inner thighs, hamstrings, quadriceps, upper calves and lower calves) and standing (hamstrings, quadriceps, and upper and lower calves). See Table 2 MS Spasticity: Take Control Stretches.

#### Control intervention

The control intervention is also two 2-h classes one week apart. This intervention is structured similarly to STC, starting with introductions, an icebreaker activity and review of the group rules. The first class continues with discussion of definitions and positions to reduce spasticity, a break, and demonstration and practice of a few of the exercises in the brochure and concludes with preparing for the next class of group exercise instruction. The second class is primarily practicing the remaining exercises described and illustrated in the brochure so participants will know proper technique when on their own. The control group also begins doing and recording daily home practice the day following the second class.

The control intervention uses the NMSS brochure *Stretching for People with MS: An Illustrated Manual* instead of STC to guide the classes [26]. The title with ‘stretching’, implies this brochure includes a set of stretching exercises and the descriptions of spasticity imply doing the exercises will manage spasticity giving this information face validity. However, most of the exercises are range of motion, strengthening and balance/coordination exercises along with several resting positions. The sitting heel cord stretch is the only exercise labeled as a stretch. The NMSS has provided this or similar information for over 30 years for free to PwMS via the NMSS or their neurologist’s office. This information was developed and has since been modified and updated by physical therapists. We feel this is an appropriate control intervention because, in our experience, PwMS who ask their healthcare providers about appropriate exercise for MS, are given this brochure because, for many years, it was the only free MS-specific exercise guidance available.

### Intervention fidelity

Facilitators were randomized to their programs by the study statistician and are trained on delivery of their respective intervention by Hugos who is a physical therapist. Facilitators complete checklists following each class confirming adherence to the schedules and topics. Retraining to ensure fidelity of the exercise instruction occurs approximately every six months during the intervention period. Facilitators are not involved in any outcome visits.

### Outcome visits

The primary endpoint of the study is after one month of home stretching that is started after the second class of exercise instruction and the secondary endpoint is after six-months of home stretching. The same outcome questionnaires (approximately 60 min) and timed walking tests are administered at the baseline visit and at each outcome visit with assessors blind to treatment allocation. Any medication changes are recorded for potential impact on study results.

### Primary outcome measure

The primary outcome is self-reported impact of spasticity measured with the MSSS. The MSSS is an 88-item self-report questionnaire, with each question answered on a 1 (not bothered at all) to 4 (extremely bothered) scale resulting in total scores ranging from 88 to 352. All questions begin with “As a result of your *spasticity*, how much in the past two weeks have you been bothered by:” and end with a specific symptom. These symptoms are organized into eight subscales in clinically relevant areas: spasticity specific symptoms (Muscle stiffness, Pain and discomfort, and Muscle spasms), areas of physical functioning (Activities of daily living, Walking, and Body movements), Emotional health and Social functioning. The MSSS was developed as a patient-focused measure to capture patient experience and perception of the impact of spasticity in MS with day-to-day symptoms and during functional activities. The MSSS total and subscale scores are valid and reliable, patient-focused measures of the *impact* of spasticity in PwMS. The MSSS highlights the complexity of the seemingly one-dimensional concept of spasticity. Extensive testing, analysis and evaluation supported the need for the eight scales to cover the breadth of the problem. The measure creators acknowledge that this new tool contributes little to improve understanding between this self-assessment and objective clinical findings, but their goal was to create a useful tool to move the field forward for assessing patient status and quality of life [23, 40]. The MSSS takes about 10 min to complete.

### Secondary spasticity-related outcome measure

The Numeric Rating Scale for Spasticity (NRSs) is used as a secondary spasticity-related outcome measure. The NRSs is a valid and reliable self-report measure of the severity of spasticity [24]. The NRSs, like the MSSS, was developed to capture information from the patient’s perspective. The NRSs is performed by asking the participant to mark on a horizontal row of numbers from 0 to 10 how severe their spasticity is, with 0 at the left end of the line being no spasticity and 10 at the right end of the line indicating the worst possible spasticity. The test-retest reliability of the NRSs is better than the AS (ICC 0.83 vs 0.53) and its validity is supported by a consistent association with Patient Global Impression of Change (PGIC) scores. 29.5% improvement on the NRSs was associated with “much improved” or better on the PGIC, consistent with the widely used Numeric Rating Scale for pain showing 30% improvement is clinically important. The 18% Minimal Clinical Important Difference (MCID) in the NRSs was also consistent with 10–20% improvement in pain recognized as the MCID for pain [24]. The NRSs takes about 2–3 min to complete.

### Other secondary outcome measures

The Modified Fatigue Impact Scale (MFIS) is used to measure impact on fatigue. The MFIS is a 21-item self-report questionnaire, with each question scored 0 (never) to 4 (always) to measure the frequency of fatigue-related problems over the prior four weeks and provides a total score and scores for three subscales: physical, cognitive, and psychosocial [27]. The MFIS takes about 5 min to complete.

The PROMIS Short Form 8a is used to measure emotional distress/depression. The PROMIS Short Form 8a is an 8-item self-report questionnaire with each question scored from 1 (never) to 5 (always) to measure negative mood (sadness, guilt), views of self (self-criticism, worthlessness), social cognition (loneliness, interpersonal alienation), and decreased positive affect and engagement (loss of interest, meaning, and purpose) over the past seven days [28]. The PROMIS Short Form 8a takes about 2–3 min to complete.

The Pittsburgh Sleep Quality Index (PSQI) is used to measure sleep quality. The PSQI has 19 self-rated items that are combined to form seven “component” scores. Each item is scored 0 (no difficulty) to 3 (severe difficulty) to measure the quality of sleep during the past month. The seven component scores are then added to yield one “global” score, with a range of 0 (no difficulty) to 21 (severe difficulties in all areas). Scores lower than 5 are associated with good quality sleep [29]. The PSQI takes about 5 min to complete.

The MS Impact Scale-29 (MSIS) is used to measure the impact of MS on day-to-day life. The MSIS is a 29-item self-report questionnaire, with each question scored 0 (not at all) to 5 (extremely) to measure the impact on daily activities as a result of MS over the previous two weeks [30]. The MSIS takes about 5 min to complete.

The Multiple Sclerosis Walking Scale-12 (MSWS) is used to measure the impact of MS on walking. The MSWS is a 12-item self-report questionnaire, with each question scored 0 (not at all) to 5 (extremely) to measure limitations in walking ability as a result of MS over the previous two weeks [31]. The MSWS takes about 5 min to complete.

The Timed Up and Go (TUG) is an examiner-administered measure of the time it takes to rise from a chair, walk 10 ft, turn around, and walk back to the chair, turn around, and sit down at their normal, comfortable walking pace. The better of two trials is used [32]. The TUG takes less than 5 min to complete.

The Timed 25 Foot Walk (T25FW) is an examiner-administered measure of the time it takes to walk 25 ft as quickly as possible but safely. Two trials are averaged [33]. The T25FW takes less than 5 min to complete.

Daily diaries are used to measure adherence to the exercise recommendations; collect any adverse events, specifically, any problems participants may have as a result of doing the exercises, and falls.

Diaries are organized with one week per page and subjects are given six weeks of diary pages at a time. Subjects are asked to fill in the date and record the number of minutes they spend doing their assigned exercises each day including zero “0” if no stretches are done. Subjects are instructed to only include the exercises they learned in their program and not to include any other exercises they do. As part of participation, we ask subjects to do the exercises they are taught but it is up to them if they want to continue with any other exercises.

Adverse events will be reviewed by the PI and rated for severity and determination whether they are study related. Any adverse events will be acted upon as needed and specified by the IRB.

Systematic collection of problems participants had doing the exercises or as a result of the exercises will allow reporting of this data. The diaries also collect number of daily falls for any reason during the six-month follow up period. Spasticity contributes to falls in people with MS so recording falls sustained during the study will help identify if the exercises impact fall frequency. Falls are counted daily. If diaries are not received, participants are called and sent a letter about returning diary pages when future diary pages are mailed to them.

### Steps to minimize drop out

Participants are compensated for their time and travel ($25 each for the baseline and the two group instruction visits and $50 for the one- and six-month follow-up visits for a total of $175). Study coordinators or facilitators contact participants by telephone one to two work days before each scheduled study activity, and as needed for questions or concerns, until participants exit the study. We have used this approach successfully in the past to retain participants [25, 36–38].

### Data management and analysis

Data from all questionnaires and diaries are entered directly by subjects using online data entry for remote baseline and/or outcome data collection or transferred from participant completed paper documents by study staff into Research Electronic Data Capture (REDCap) and REDCap Survey, secure on-line applications [41]. The PI, study statistician and outcome assessors are blind to treatment assignment throughout the trial. Once the last participant has exited the trial, the database will be finalized and “locked.” All personal health information is removed before sending data for analysis. All primary and secondary data analyses are performed on the database with allocation coded as A and B. Unblinding will only occur after the primary and secondary data analyses are complete. This minimal risk study does not have a Data Management Committee and all aspects of study management are overseen by the PI. Participants’ study information will not be released outside of the study without the written permission of the participant, except as necessary for the oversight agency.

The data from this study are maintained in a data repository at VAPORHCS. Interested investigators may access this data for IRB-approved research or activities preparatory to research by making a request to the repository director (Dr. Cameron) and completing a Data Use Agreement with VAPORHCS. Study documents that do not contain subject data, such as protocol and statistical analysis plan, may also be shared upon request.

Trial results will be authored by study team members and disseminated at professional meetings and via publication in peer-reviewed journals.

### Power analysis and sample size

Based on our pilot trial with 38 people, the mean of percent change in MSSS scores was − 2% for the usual care (UC) group with unguided use of the NMSS brochure and − 14% for the STC group. Both had a standard deviation of 0.17. We believe a 3-fold improvement in percentage change for the STC group vs UC group (i.e. 12% decrease in the STC group vs. 4% decrease in the UC group) is clinically significant. Therefore, we need 100 participants in each group (200 in total) to achieve 80% power using a 2-sided t-test at 5% significance level assuming the standard deviation of the percentage change from baseline for all participants is 0.2. Using 80% power and projecting for 10% drop-out rate, we will recruit 220 participants total (110 per group). We based the 10% drop-out on our previous experience of 95% retention in the spasticity pilot study and 94% retention in our 200 subject fatigue study and evidence that 20% attrition is considered good for self-management interventions [25, 37, 38, 42–44].

### Statistical analysis of outcomes

Summary statistics of outcome scores will be calculated and quality control assessments will be completed. Missing data will be treated conservatively. Intent-to-treat (ITT) and per protocol analyses will be used to test the hypotheses. Expecting few missing data and assuming no difference, ITT will be reported. All tests will be two-sided and *p* = 0.05 will be used to determine significance.

Univariate statistical analysis will be conducted for all primary and secondary endpoints within each group. For continuous measurements, we will use two-sample t test (for standardized measurements) or Wilcoxon rank sum test to compare the changes from baseline between the two groups, and use paired t test or signed test to determine whether there is a significant change from baseline within each group. For categorical variables, we will use Chi-square test or Fisher’s exact test, as appropriate, for between group comparisons, and McNemar’s test for within group comparisons. Weighted Cohen’s Kappa will be computed to determine the level of agreement in similar measurements obtained with different approaches from the same participant. A mixed effect model will be used to assess whether the changes over time differ between groups. We will use linear regression models to evaluate for potential mediators and confounders for the impact of the intervention on outcomes. Multiple linear regression models may be fitted to compare the measurement changes between two groups if one or more demographic variables need to be controlled.

## Discussion

This study is the first fully powered study to evaluate the benefits, risks and adherence to education and stretching instruction for MS spasticity management. Education and stretching exercises are commonly recommended from the onset of spasticity symptoms to be done daily for the duration of spasticity symptoms and are the current standard of care, but there is no objectively researched evidence of benefit.

This study compares the impact of two MS education and exercise programs on spasticity in a randomized controlled trial. One program, the stretching intervention, focuses on MS spasticity education and stretches and the other, the control intervention, discusses general MS-related information, terminology, positioning, and exercises that focus on range of motion, balance, coordination and strengthening. Both programs have facilitators and are delivered to groups of ambulatory people with MS and self-reported lower extremity spasticity. Participants have the opportunity to try the exercises in the group classes and are then instructed to do them daily at home for the following 6 months and record the time spent doing the exercises in daily diaries.

Spasticity is very problematic for those living with it and available treatments are generally seen as inadequate or insufficient. The NARCOMS registry analysis (*n* = 20,969) found ~ 6500 people with minimal spasticity (spasticity is noticeable but does not interfere with activities), ~ 4000 people with mild spasticity (spasticity forces changes in activities once a week or less), ~ 3600 people with moderate spasticity (spasticity interferes with activities several times per week), ~ 2700 people with severe spasticity (spasticity forces modification in activities every day), and ~ 800 people with total spasticity (spasticity prevents activities every day). In total, 84% of these registrants were dealing with some level of spasticity every day [2]. The German MObility ImproVEment (MOVE) study (*n* = 414) reported “disturbing symptoms” of stiffness in 74% and mobility restrictions in 66% of participants, “impairment of daily activities” in 10% of those with mild spasticity and up to 85% of those with severe spasticity, and treatment at enrollment with drugs and rehabilitation was common, with 55% of people receiving medications and 78% receiving treatment with physiotherapy. The proportions increased with increasing severity of spasticity. Finally, both physicians (41%) and patients (35%) were partially dissatisfied or dissatisfied with results from available anti-spasticity medications [45]. Bethoux and Marrie surveyed 15,000 NARCOMS participants and found 35% were moderately to greatly bothered by stiffness, spasms or pain. Most were treated for their spasticity but under half were satisfied with their treatment [46].

It is not uncommon to find physical therapy (or “skilled rehabilitation”) recommended as a treatment for spasticity but the physical therapy treatment is actually poorly defined [2, 21, 22, 47]. The core component of a skilled rehabilitation program for spasticity is stretching, in addition to positioning, orthotics, casting and other interventions, with little evidence supporting any of them [3, 4, 12]. Often orthotics and casting are designed to provide a prolonged stretch. Stretching is typically designed as a one to two times daily home exercise program that patients and their physicians and therapists are hopeful will counteract the continuous – present 24 h per day - contracting - muscle shortening - stimulus of spasticity. To date there is one small study of baclofen with 30 PwMS and spasticity in the quadriceps that found significant improvement on the MAS with oral baclofen compared to placebo and compared to placebo with stretching exercises. Adding stretching exercises to baclofen treatment was associated with a trend for additional benefit [19]. This was primarily a study of the benefit of oral baclofen on spasticity in one muscle group. Spasticity is often more generalized, affecting more than one muscle group or even multiple entire limbs. A systematic review on stretching for spasticity found some evidence for immediate effects of one stretching session, but the authors concluded that it remained unclear how long the effects lasted and if there were any long-term impacts [20]. Systematic reviews investigating the effects of stretching for prevention or treatment of contractures did not find benefit of stretching for prevention or treatment of contractures in neurologic and other conditions if performed for less than seven months and found no studies reporting treatment longer than seven months [21, 22]. An overview of systematic reviews for non-pharmacological interventions for spasticity in adults found low quality evidence for rehabilitation programs, including stretching; physical activity programs with stretching, and very low evidence for passive movement [48]. Another review, specifically of physical therapy interventions for spasticity reported the best quality evidence for beneficial effects for exercise therapy, especially robot gait training, and outpatient exercise programs, both requiring supervision or 1:1 treatment, but concluded no firm conclusions can be drawn on the effect of physical therapy interventions on overall spasticity [49].

### Strengths

This will be a large study, once fully enrolled, with 200 ambulatory subjects with MS and self-reported spasticity. All subjects, both those in the active group and those in the control group, will receive an intervention with comparable time and attention and expectations of doing the exercises daily for the following six months. In addition to spasticity outcomes, we are carefully collecting information on changes in spasticity or MS disease modifying medications, exercise adherence with the number of minutes spent doing the exercises daily for the six months of follow-up, problems and adverse events experienced by the participants doing the exercises, and falls. Few studies systematically collect and report adverse events or problems related to the exercises, or collect and report exercise adherence, as we plan to do. Activities and quality of life are affected by spasticity so we are collecting information on many of these with our secondary outcomes. The programs will be offered weekdays at various day and evening times in urban and suburban locations within the VAPORHCS Federalwide Assurance area (where VAPORHCS is the presiding IRB).

### Limitations

Potential limitations of this study include the use of self-reported spasticity, rather than clinician or instrument-based measures of spasticity, as the spasticity-related entry criterion and primary and secondary outcome; potential confounding from unmeasured factors, and generalizability. We feel using of self-reported spasticity as the primary outcome measure outweighs the limitations of the alternatives to capture patients’ lived experiences of spasticity [18]. Measuring range of motion may have captured a consequence of spasticity but not the experience of spasticity. Careful attention to the descriptions used in the NARCOMS description of spasticity should minimize people confounding abnormal sensations with spasticity. We may miss effects of botulinum injections that are dependent on the cycle of the injections. As with any study, there may be confounding from unmeasured factors (botulinum injections, for instance, and other lifestyle interventions discussed in STC such as massage and acupuncture) which should be controlled by randomization. Generalizability of the results of this study may be limited by the study being carried out in a region of the United States with low racial and ethnic diversity, use of few facilitators and not being powered to compare effects between facilitators, and by only including subjects who can walk. An additional limitation is that we are asking participants to do their assigned exercises daily for six months when current clinical practice recommends daily stretching as needed for the rest of one’s life. Therefore, this study will not provide information on the effects of stretching for spasticity beyond the medium term of six months.

### Implications/future directions

If the stretching intervention is found to be more effective than the control intervention, the results of this study will be the first compelling evidence that spasticity education and stretching exercises help reduce the impact of spasticity in PwMS. Presently, patients are asked to do daily stretching through the duration of spasticity symptoms without clear evidence supporting a benefit from the investment in time and energy. This study may support that daily stretching is worthwhile, at least for six months. Further research is needed to investigate adherence to and effects of stretching beyond six months.

If effective, STC also has potential for broader application. STC could be delivered in a group setting to increase access to PwMS. It could be translated into other languages. STC could be adapted for on-line delivery to participants unable to attend group classes due to distance or other time commitments. STC could also be adapted to serve people with other conditions that cause spasticity such as stroke, traumatic brain injury, spinal cord injury and cerebral palsy.

## Data Availability

Upon request.

## Abbreviations

AS: Ashworth Scale
CAM: Complementary and alternative medical approaches
DVD: Digital video disc
EMR: Electronic medical record
FDA: Food and Drug Administration
ICC: Intraclass correlation coefficient
IRB: Institutional Review Board
ITT: Intent to treat
MAS: Modified Ashworth Scale
MCID: Minimal Clinical Important Difference
MFIS: Modified Fatigue Impact Scale
MOVE: German study on MObility ImproVEment
MS: Multiple sclerosis
MSIS: MS Impact Scale-29
MSSS: MS Spasticity Scale-88
MSWS: MS Walking Scale-12
NARCOMS: North American Research Committee on MS
NMSS: National Multiple Sclerosis Society
NRSs: Numeric Rating Scale for Spasticity
NW: Northwest
OHSU: Oregon Health & Science University
PDDS: Patient Determined Disease Steps
PGIC: Patient Global Impression of Change
PROMIS: Patient-Reported Outcomes Measurement Information System
PSQI: Pittsburgh Sleep Quality Index
PwMS: people with MS
REDCap: Research Electronic Data Capture
STC: MS Spasticity: Take Control
SW: Southwest
THC: Tetrahydrocannabinol
T25FW: Timed 25 Foot Walk
TUG: Timed Up and Go
UC: Usual care
US: United States
VA RR&D: Veteran Administration Rehabilitation Research and Development
VAPORHCS: Veteran Administration Portland Health Care System
